# A Review Study on Macrolides Isolated from Cyanobacteria

**DOI:** 10.3390/md15050126

**Published:** 2017-04-26

**Authors:** Mengchuan Wang, Jinrong Zhang, Shan He, Xiaojun Yan

**Affiliations:** 1 School of Marine Sciences, Laboratory of Marine Natural Products, Ningbo University, Ningbo 315211, China; mengchuanwang1@163.com (M.W.); heshan@nbu.edu.cn (S.H.); 2 Key Laboratory of Applied Marine Biotechnology of Ministry of Education, Ningbo University, Ningbo 315211, China; yanxiaojun@nbu.edu.cn

**Keywords:** cyanobacteria, macrolides, secondary metabolites, bioactivity

## Abstract

Cyanobacteria are rich sources of structurally-diverse molecules with promising pharmacological activities. Marine cyanobacteria have been proven to be true producers of some significant bioactive metabolites from marine invertebrates. Macrolides are a class of bioactive compounds isolated from marine organisms, including marine microorganisms in particular. The structural characteristics of macrolides from cyanobacteria mainly manifest in the diversity of carbon skeletons, complexes of chlorinated thiazole-containing molecules and complex spatial configuration. In the present work, we systematically reviewed the structures and pharmacological activities of macrolides from cyanobacteria. Our data would help establish an effective support system for the discovery and development of cyanobacterium-derived macrolides.

## 1. Introduction

Cyanobacteria, also known as blue-green algae, including cyanobacteria from terrestrial, freshwater and marine ecosystems, are a group of ancient photosynthetic prokaryotes. As defensive chemicals, structurally-diverse secondary metabolites from cyanobacteria have been proven to greatly contribute to successful survival and reproduction of cyanobacteria in changing, complex and diverse environments during the long-lasting evolutionary process [1]. At present, hundreds of compounds with important bioactivities have been isolated from terrestrial or marine cyanobacteria [2]. Macrolides are a class of important bioactive compounds, which are commonly found in marine organisms, including cyanobacteria [3]. Some marine macrolides are promising candidates for future applications in medicine. For example, bryostatin-1 shows potent antitumor activity in phase I cancer clinical trials [4]. Macrolide antibiotics, such as erythromycin and polyene macrolides, have been employed for widespread application of severe bacterial infections [5]. Structurally-diverse macrolides from cyanobacteria often contain unique and unusual substituents, including chlorinated residues, thiazole residues [6] or pyran residues [7]. Macrolides usually exhibit potent antitumor or antibacterial activities [8]. In addition, cyanobacteria have great potentials as sustainable sources for production of bioactive macrolides because of their rapid growth, genetic tractability and cultivable property [2]. Although cyanobacteria possess cultivable properties similar to those of microorganisms, cyanobacteria have attracted far less attention than microorganisms.

In the present review paper, we systematically summarized the structures and bioactivities of macrolides isolated from cyanobacteria, and over 50 references were cited. Up to the end of 2016, a total of 64 macrolide compounds have been isolated from cyanobacteria, including 49 macrolides from marine cyanobacteria and 15 macrolides from terrestrial cyanobacteria, most of which are mainly from *Lyngbya*, *Scytonema* and *Oscillatoria*. It has been reported that most of these cyanobacterium-derived macrolides possess several noticeable bioactivities, including antitumor, antibacterial, antimalarial and toxicity to animals. This review summarizes new macrolides derived from cyanobacteria, providing useful information in the further discovery of novel cyanobacterial macrolides.

## 2. Anti-Neoplastic Property of Cyanobacterium-Derived Macrolides on Different Cell Lines

**Figure 1:**
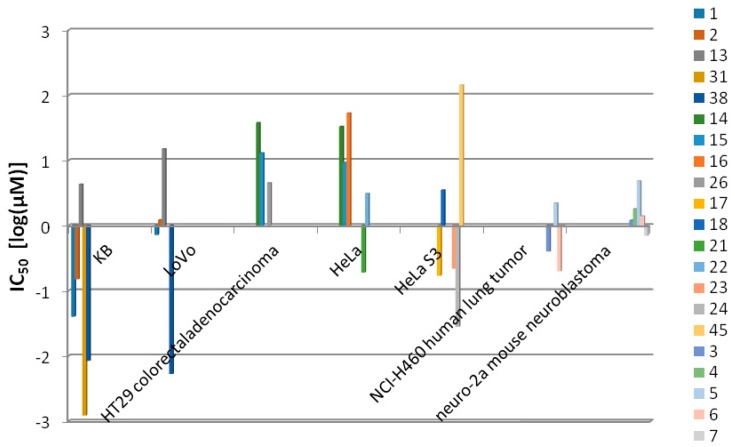
Anti-neoplastic profiling results for cyanobacterium-derived macrolides on different cell lines. Data are represented as IC_50_ [log(μM)].

Nitrogen mustard has been used in the treatment of lymphoma cancer since 1940s, and more than 100 anti-cancer drugs are widely used in the world. Until now, natural products have largely contributed to cancer therapy and become an indispensable source for the development of innovative anti-cancer drugs [9]. Most macrolides from cyanobacteria display significant cytotoxicity to cancer cells. Cyanobacteria of the genera *Symploca*, *Lyngbya*, *Scytonema* and *Oscillatoria* are important sources of anti-cancer macrolides. Cyanobacterium-derived macrolides reported to have anti-neoplastic effects on different cell lines are given in Figure 1 and Table 1.

**Figure 2:**
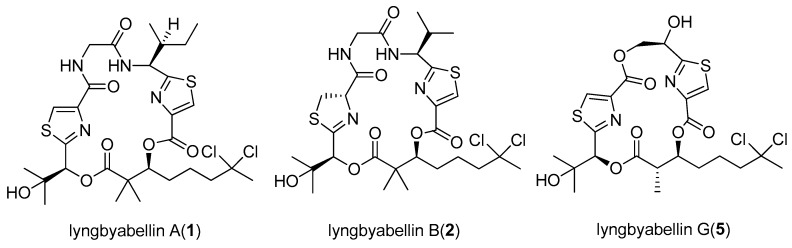

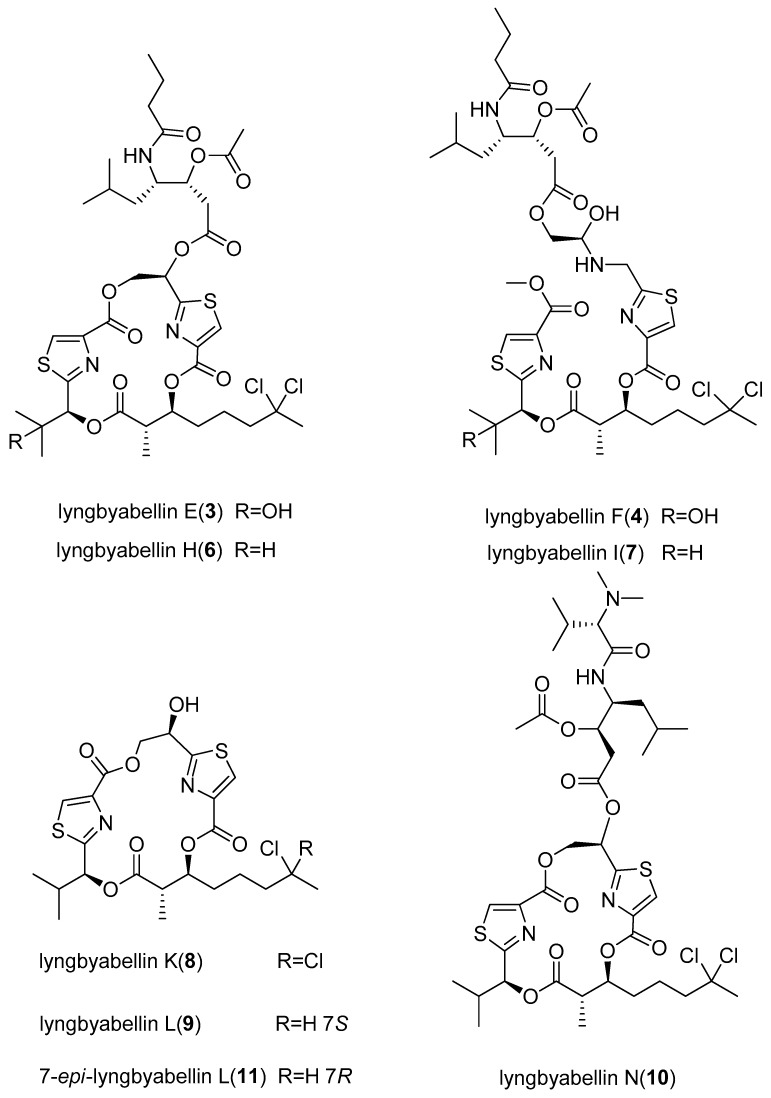
Chemical structures of Compounds **1**–**11**.

A series of cytotoxic marine cyanobacterial metabolites, named lyngbyabellins (**1**–**11**) possessing thiazole residues and chlorine substituents, have been isolated from the cyanobacterial genus *Lyngbya* (Figure 2). Isolated from the marine cyanobacterium *Lyngbya majuscula* collected from Guam, lyngbyabellin A (**1**) exhibits potent in vitro cytotoxicity against human carcinoma of nasopharynx Cell (KB cells) and LoVo cells with IC_50_ values of 0.03 and 0.50 µg/mL, respectively [6]. The analog of lyngbyabellin A (**1**), lyngbyabellin B (**2**), was isolated from the same strain of *Lyngbya majuscula*. Compared with lyngbyabellin A (**1**), lyngbyabellin B (**2**) is slightly less cytotoxic to KB and LoVo cells with IC_50_ values of 0.10 and 0.83 µg/mL, respectively [10]. Five analogs of lyngbyabellin A (**1**), including lyngbyabellins E-I (**3**–**7**), are produced from the same strain of *Lyngbya majuscula* harvested in Papua New Guinea. To the best of our knowledge, lyngbyabellins E-I (**3**–**7**) have potent in vitro cytotoxicity against human lung tumor (NCI-H460) and mouse neuroblastoma (neuro-2a) cells. Lyngbyabellin E (**3**) and lyngbyabellin H (**6**) display significant cytotoxicity to NCI-H460 (LC_50_ values of 0.4 and 0.2 µM, respectively) and neuro-2a cells (LC_50_ values of 1.2 and 1.4 µM, respectively). Lyngbyabellins F-G (**4**–**5**) and lyngbyabellin I (**7**) are slightly less cytotoxic to NCI-H460 (LC_50_ values of 1.0, 2.2 and 1.0 µM, respectively) and neuro-2a cells (LC_50_ values of 1.8, 4.8 and 0.7 µM, respectively) [11]. The marine cyanobacterium *Moorea bouillonii* (formerly *Lyngbya bouillonii*) collected from Palmyra Atoll affords four analogs of lyngbyabellin A (**1**), lyngbyabellins K (**8**), L (**9**), N (**10**) and 7-*epi*-lyngbyabellin L (**11**). Lyngbyabellin N (**10**) shows variable cytotoxicity to H-460 human lung carcinoma (IC_50_ = 0.0048–1.8 μM) and potent in vitro cytotoxicity against the HCT116 colon cancer cell line (IC_50_ = 40.9 ± 3.3 nM). This result could perhaps be explained by the solubility problem of lyngbyabellin N (**10**). The nitrogen-containing side chain (leucine statine residue) of lyngbyabellin N (**11**) may be the basic structural feature for its cytotoxic activity [12].

**Figure 3:**
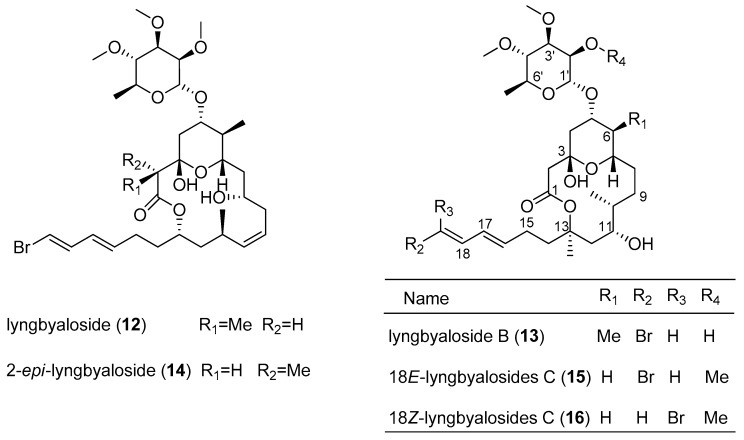
Chemical structures of Compounds **12**–**16**.

Several 16-membered glycoside macrolides, termed lyngbyalosides, are produced from various species of the cyanobacterial genus *Lyngbya* (Figure 3). The marine *Lyngbya bouillonii*, collected from Laing Island, afford lyngbyaloside (**12**) [8]. Lyngbyaloside B (**13**), isolated from marine cyanobacterium *Lyngbya* sp., which was collected from Palaua, shows weak cytotoxicity against KB cells and LoVo cells with IC_50_ values of 4.3 and 15 µM, respectively [13]. The total synthesis of lyngbyaloside B (**13**) has been reported by Fuwa et al. [33]. Three analogs of lyngbyaloside (**12**), including 2-*epi*-lyngbyaloside (**14**), 18*E*-lyngbyaloside C (**15**) and 18*Z*-lyngbyaloside C (**16**), were isolated from the marine cyanobacterium *Lyngbya bouillonii*, collected from Apra Harbor, Guam. Cytotoxicity assays revealed that these macrolides possess weak to moderate cytotoxicity against the human colorectal adenocarcinoma cell line HT29 and HeLa cervical carcinoma cells. 18*E*-lyngbyaloside C (**15**) is more cytotoxic toward HT29 colorectal adenocarcinoma and HeLa cervical carcinoma cells (IC_50_ values of 13 and 9.3 µM, respectively) than 2-*epi*-lyngbyaloside (**14**) (IC_50_ values of 38 and 33 µM, respectively). 18*E*-Lyngbyaloside C (**15**) is approximately five-fold more cytotoxic than 18*Z*-lyngbyaloside C (**16**) (IC_50_ values of >100 µM and 53 µM, respectively) [14]. The total synthesis of lyngbyaloside C has also been accomplished [34].

**Figure 4:**
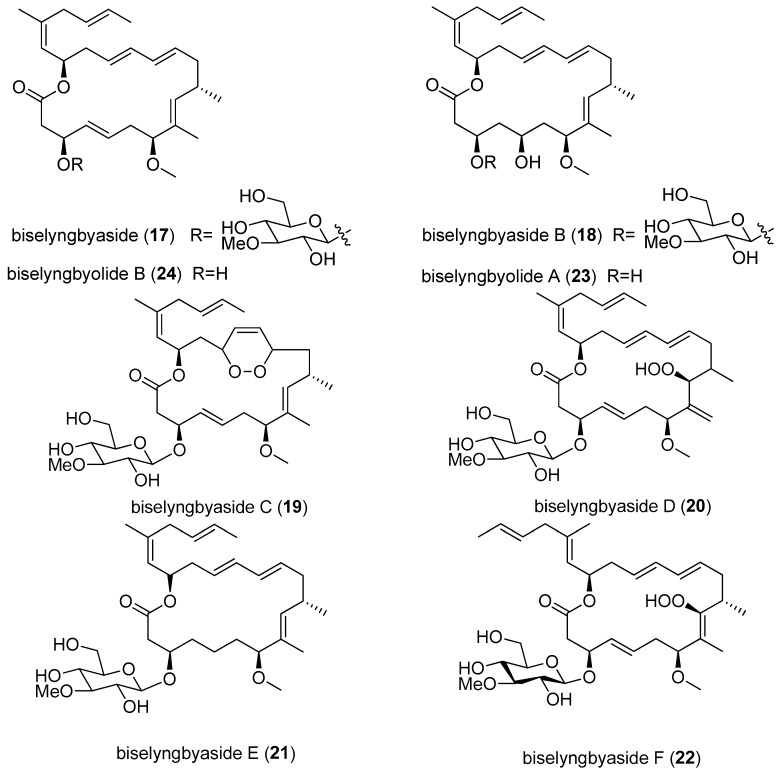
Chemical structures of Compounds **17**–2**4**.

Another distinct class of 18-membered ring glycoside macrolides has been isolated from the cyanobacterial genus *Lyngbya* (Figure 4). Biselyngbyaside (**17**) was discovered through a bioassay-guided screening for cytotoxic compounds from cyanobacterium *Lyngbya* sp. collected from Okinawa Prefecture, Japan. Biselyngbyaside (**17**) shows a broad spectrum of cytotoxicity against human solid tumor cell lines, especially for HeLa S_3_ cells with an IC_50_ value of 0.1 μg/mL [15], and its total synthesis was completed [35]. Extensive efforts toward finding cytotoxic natural products have resulted in the isolation of three analogs of biselyngbyaside (**17**), named biselyngbyasides B–D (**18**–**20**), from the marine cyanobacterium *Lyngbya* sp. Biselyngbyaside B (**18**) displays significant cytotoxicity against HeLa S_3_ and HL60 cells (IC_50_ values of 3.5 and 0.82 µM, respectively, using thapsigargin as a positive control drug). In addition, biselyngbyasides B-D (**18**–**20**) induced apoptosis of cancer cells by inhibiting calcium influx into the endoplasmic reticulum and increasing the concentration of intracellular calcium [16]. Two analogs of biselyngbyaside (**17**), biselyngbyasides E (**21**) and F (**22**), were isolated from the marine cyanobacterium *Lyngbya* sp. collected from Ishigaki Island, Japan. In vitro cell cytotoxicity assays showed that biselyngbyaside E (**21**) has higher cytotoxicity against HeLa and HL60 cells (IC_50_ values of 0.19 and 0.071 µM, respectively) than biselyngbyaside F (**22**) (IC_50_ values of 3.1 and 0.66 µM, respectively). Based on the trisubstituted olefin geometry, the presence and absence of the sugar moiety are crucial for the biological activities [17].

Like a cytotoxic biselyngbyaside-related macrolide, biselyngbyolide A (**23**) was isolated from the marine cyanobacterium *Lyngbya* sp. harvested from Tokunoshima Island, Japan. Biselyngbyolide A (**23**) shows strong cytotoxicity against HeLa S_3_ cells and HL60 cells with IC_50_ values of 0.22 and 0.027 µM, respectively [18]. Biselyngbyolide B (**24**) was also isolated from the same strain of *Lyngbya* sp. and displays significant inhibitory effects on growth of HeLa S_3_ cells and HL60 cells (IC_50_ values of 0.028 and 0.0027 µM, respectively, using thapsigargin as a positive control drug). Moreover, biselyngbyolide B (**24**) has 3–100-fold more potent apoptosis-inducing activity than biselyngbyaside (**17**) [16,19].

**Figure 5:**
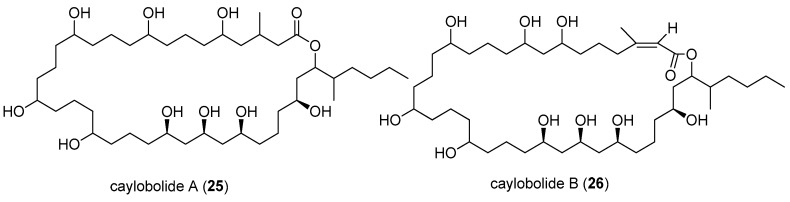
Chemical structures of Compounds **25** and **26**.

A novel 36-membered macrolactone, caylobolide A (**25**), was isolated from Bahamian cyanobacterium *Lyngbya majuscula*, which contains an unprecedented repeating unit, an adjoining pentad of 1,5-diols and a 1,3,5-triol (Figure 5). In vitro cytotoxicity assay showed that caylobolide A (**25**) possesses potent cytotoxicity against human colon tumor cells HCT-116 with an IC_50_ value of 9.9 µM [20], and its total synthesis has been accomplished [36]. Caylobolide B (**26**) was isolated from the marine cyanobacterium *Phormidium* spp. collected from Key West, Florida, and it exhibits strong cytotoxicity against HT29 colorectal adenocarcinoma (IC_50_ value of 4.5 µM) and HeLa cervical carcinoma cells (IC_50_ value of 12.2 µM) [21].

**Figure 6:**
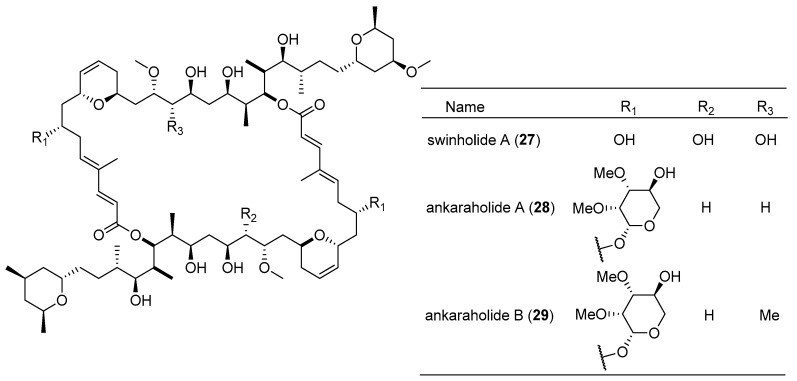
Chemical structures of Compounds **27**–**29**.

Swinholide A (**27**), originally isolated from the marine sponge *Theonella swinhoei*, was isolated from the marine cyanobacterium *cf*. *Symploca* sp. collected from Fiji and was found to strongly inhibit the growth of several tumor cell lines with IC_50_ values ranging from 0.37 nM to 1.0 μM [22]. Two swinholide-based glycosylated macrolides, named ankaraholides A,B (**28**,**29**), were isolated from two field collections of marine cyanobacteria (Figure 6). Ankaraholide A (**28**) exhibits strong antiproliferative activity against NCI-H460, Neuro-2a and MDA-MB-435 cell lines with IC_50_ values of 119, 262 and 8.9 nM, respectively. Ankaraholide A (**28**) inhibits proliferation of A-10 cells by inducing complete loss of the filamentous (F)-actin during the cell extending process when the concentration of ankaraholide A (**28**) reaches 30 nM [22].

**Figure 7:**
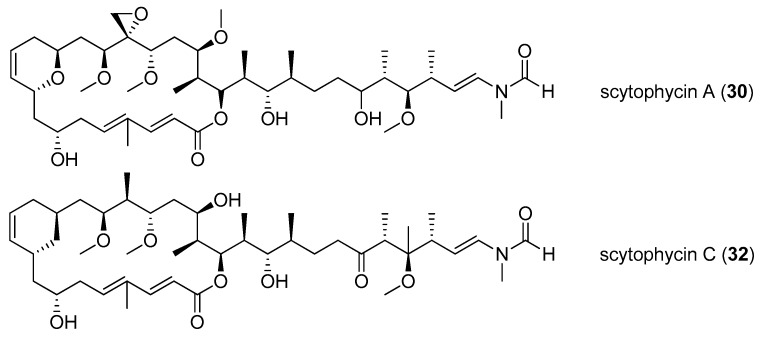

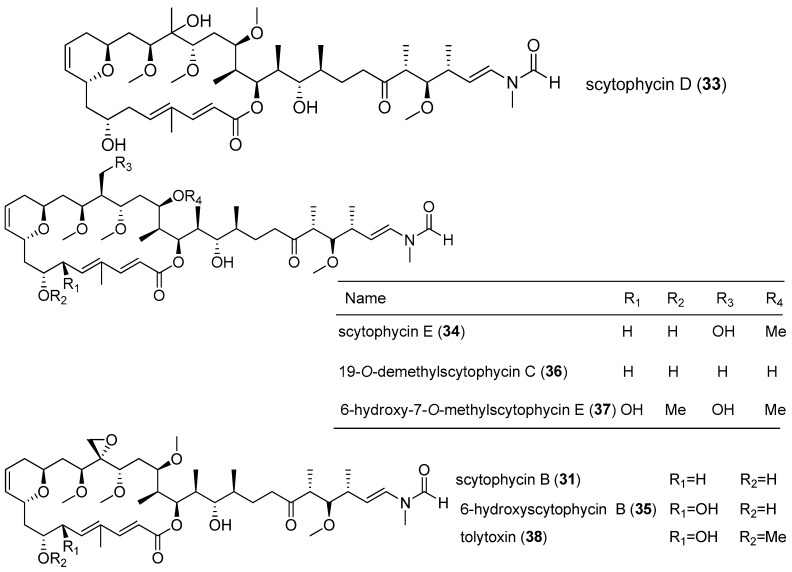
Chemical structures of Compounds **30**–**38**.

A family of potent cytotoxic natural products, scytophycins A–E (**30**–**34**), was isolated from the terrestrial cyanobacterium *Scytonema pseudohofmanni* [37]. Scytophycins A (**30**) and B (**31**) display significant cytotoxicity against KB cells (IC_50_ value of 1 ng/mL), while scytophycins C-E (**32**–**34**) are less cytotoxic to KB cells (IC_50_ values ranging from 10 to 100 ng/mL) than scytophycin A (**30**) [23]. Total synthesis of scytophycin C (**32**) has been completed [38]. Screening of cyanobacteria leads to the discovery of three analogs of scytophycins, including 6-hydroxyscytophycin B (**35**), 19-*O*-demethylscytophycin C (**36**) and 6-hydroxy-7-*O*-methylscytophycin E (**37**) (Figure 7). These compounds (**35**–**37**) show strong inhibitory effect on the growth of KB (MIC values ranging from 1 to 5 ng/mL) and LoVo cells (MIC values ranging from 10 to 50 ng/mL) [23]. The cytotoxic tolytoxin (**38**) was isolated from terrestrial cyanobacterium *Tolypothrix conglutinata*, collected from Fanning Island [39], and displays excellent cytotoxicity against LoVo and KB cells with IC_50_ values of 8.4 and 5.3 nM, respectively [24].

**Figure 8:**
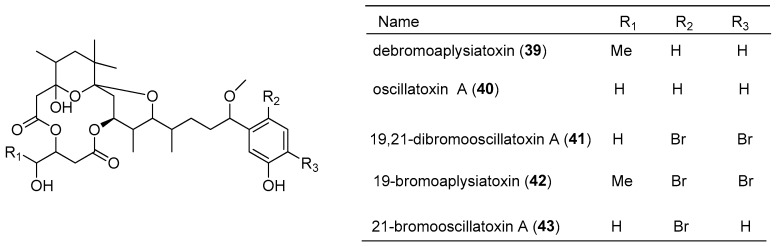

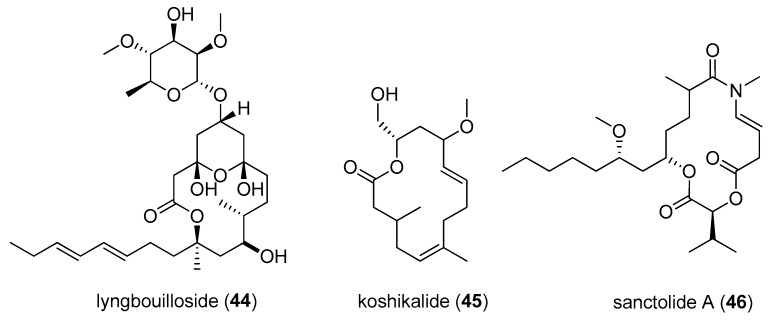
Chemical structures of Compounds **39**–**46**.

Debromoaplysiatoxin (**39**) was isolated from the marine cyanobacterium *Lyngbya majuscula*, collected from Hawaii [40], and shows potent cytotoxicity against mouse lymphocytic leukemia P-388 [25]. Four analogs of debromoaplysiatoxin (**39**), including oscillatoxin A (**40**), 19,21-dibromooscillatoxin A (**41**), 19-bromoaplysiatoxin (**42**) and 21-bromooscillatoxin A (**43**), were isolated from a mixture of marine cyanobacteria *Oscillatoria nigroviridis* and *Schizothrix calcicola* from Enewetak Island (Figure 8). These compounds (**41**–**43**) contain the same 14-membered macrocycle as debromoaplysiatoxin (**39**), but they are bromine-containing macrolides [41]. A 14-membered glycosidic macrolide, lyngbouilloside (**44**), was isolated from the marine cyanobacterium *Lyngbya bouillonii*, harvested from Papua New Guinea. It displays a modest cytotoxicity against neuroblastoma cells with an IC_50_ value of 17 µM [26]. Another 14-membered macrolide, koshikalide (**45**), was isolated from the marine cyanobacterium *Lyngbya* sp., collected from Mie Prefecture, Japan, and shows slight cytotoxicity against HeLa S_3_ cells with an IC_50_ value of 42 µg/mL [27]. In addition, the total synthesis of koshikalide (**45**) has been achieved by exploiting a novel convergent strategy [42]. A 14-membered marine macrolide, sanctolide A (**46**), containing a rare *N*-methyl enamide and a 2-hydroxyisovaleric acid, was obtained from the culture of cyanobacterium *Oscillatoria sancta*. It is cytotoxic against HT-29 and MDA-MB-435 cell lines [28], and its total synthesis was achieved [43].

**Figure 9:**
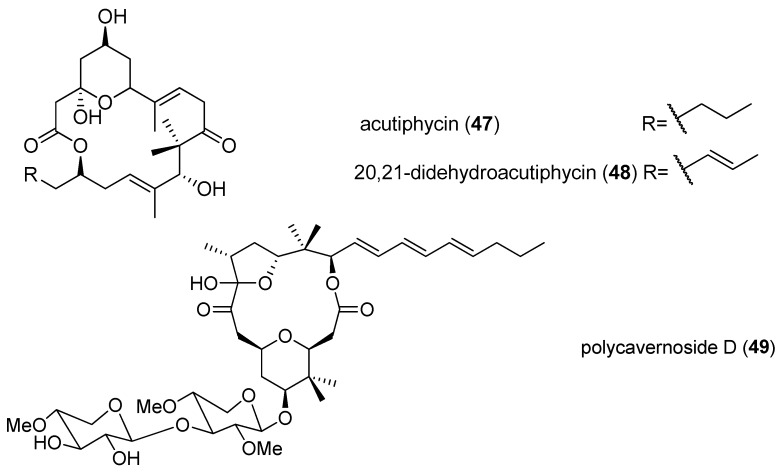

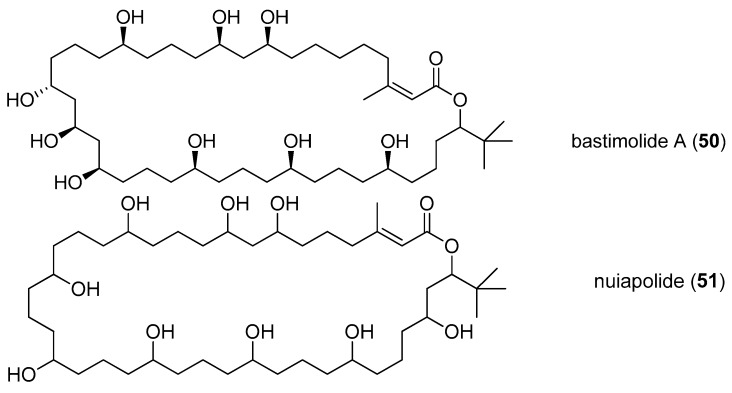
Chemical structures of Compounds **47**–**51**.

Two cytotoxic marcolides, acutiphycin (**47**) and 20,21-didehydroacutiphycin (**48**), were isolated from freshwater cyanobacterium *Oscillatoria acutissima*, collected from Manoa Valley, Oahu, and possess strong cytotoxicity against KB and NIH/3T3 cells (ED_50_ < 1 μg/mL), as well as Lewis lung carcinoma [29]. A rare marine toxin, polycavernoside D (**49**), was obtained from the marine *Okeania* sp. collected from the Caribbean (Figure 9). The discovery of polycavernoside D, for the first time, provides a conclusive proof that these lethal toxins (polycavernosides) have, in fact, a cyanobacterial origin rather than other marine organisms. Polycavernoside D (**49**) displays cytotoxicity against the H-460 human lung cancer cell line in a dose-dependent manner, with an EC_50_ value of 2.5 µM [30]. Bastimolide A (**50**), isolated from the marine *Okeania hirsuta* from Bastimentos Park, Panama, is a rare 40-membered polyhydroxy macrolide consisting of one 1,3-diol, one 1,3,5-triol, six 1,5-diols and one *tert*-butyl group. Bastimolide A (**50**) exhibits strong cytotoxicity against Vero cells with an IC_50_ value of 2.1 µM [31].

A rare 40-membered macrolactone, nuiapolide (**51**), was isolated from Niihau marine cyanobacterium. As a polyhydroxylated macrolide, nuiapolide (**51**) contains a rare *tert*-butyl carbinol residue, and it displays anti-chemotactic activity against Jurkat cells and cancerous T lymphocytes and can trigger a predominant G2/M phase shift in the cell cycle [32].

## 3. Antibacterial Activity

Some macrolides, such as erythromycin and azithromycin, have shown excellent antibacterial activity and are widely used in clinical practice of various types of bacterial infections [44]. Some macrolides from cyanobacteria also show good antibacterial activities. Cyanobacterium-derived macrolides with antimicrobial properties are listed in Table 2.

Scytophycins C–E (**32**–**34**) were isolated from the terrestrial cyanobacterium *Scytonema pseudohofmanni*, collected from Oahu, Hawaii, and were shown to exhibit weak antibacterial activity [37]. Three analogs of scytophycin C (**32**), including 6-hydroxyscytophycin B (**35**), 19-*O*-demethylscytophycin C (**36**) and 6-hydroxy-7-*O*-methylsctophycin E (**37**), were isolated from the cyanobacteria *S*. *mirabile*, *S*. *burmanicurn* and *S*. *ocellatum*, respectively. These macrolides (**35**–**37**) display antifungal activity against *Aspergillus oryzae*, *Candida albicans*, *Penicillium notatum* and *Saccharomyces cerevisiae* [23]. The cytotoxin, tolytoxin (**38**), was isolated from the terrestrial cyanobacterium *Tolypothrix conglutinata*, collected from Fanning Island [39], and was found to exhibit potent antifungal activity against various yeasts and filamentous fungi (MICs of 0.25–8 nM) [24].

**Figure 10:**
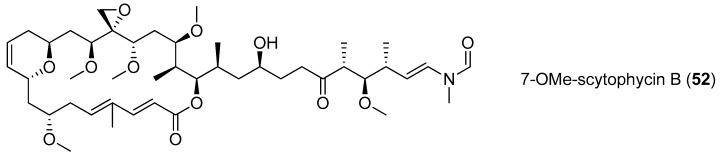

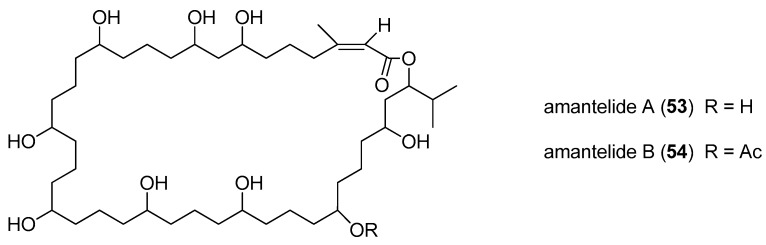
Chemical structures of Compounds **52**–**54**.

A bioactive marcolide, 7-OMe-scytophycin B (**52**), was identified from a culture of a marine cyanobacterium and was found to exhibit antifungal activity against *Candida albicans* HAMbI 484 and *Candida guilliermondii* HAMBI 257 with MIC values of 0.40 and 0.80 mM and IC_50_ values of 0.19 and 0.23 mM, respectively [45]. Two 40-membered macrolactones, amantelides A,B (**53**,**54**), are composed of a 1,3-diol and contiguous 1,5-diol units and a *tert*-butyl substituent. These compounds were isolated from a Guam cyanobacterium belonging to the family Oscillatoriales (Figure 10). Amantelide A (**53**) shows a broad spectrum of inhibitory effects on the growth of both eukaryotic and prokaryotic cells. The growth of the fungi *Lindra thalassiae* and *Fusarium* sp. is completely inhibited when the concentration of amantelide A (**53**) is 62.5 μg/mL. When the concentration of amantelide B (**54**) is 6.25 μg/mL, the growth of the fungus *Dendryphiella salina* is completely inhibited [46].

## 4. Effects of Cyanobacterium-Derived Macrolides on Animals

Toxin-producing cyanobacterial blooms are a potential health risk for other living organisms, including humans [47]. Cyanobacterium-derived macrolides show toxicity to animals, such as brine shrimp and mice. The effects of cyanobacterium-derived macrolides on fauna are described in Table 3.

The cytotoxic macrolactone, lyngbyabellin A (**1**), exhibits potent toxicity to mice in vivo trials (lethal dose of 2.4 to 8.0 mg/kg; sublethal dose of 1.2 to 1.5 mg/kg) [6]. Tolytoxin (**38**) is highly toxic to mice with a sublethal dose (ip) of 1.5 mg /kg [24].

**Figure 11:**
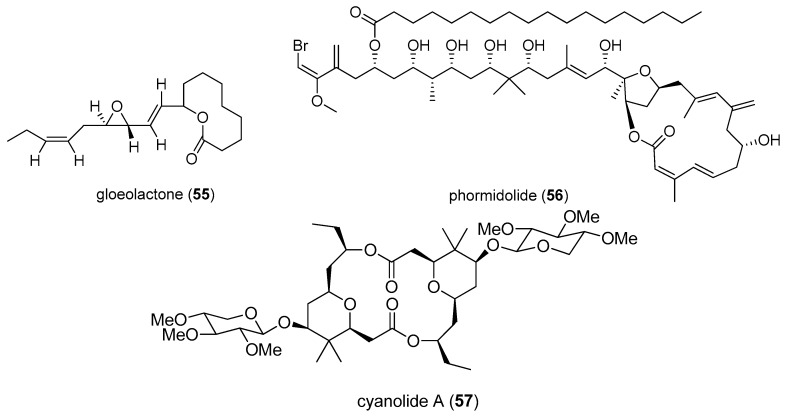
Chemical structures of Compounds **55**–**57**.

A 14-membered macrolide, sanctolide A (**48**), shows high toxicity toward the brine shrimp with an LC_50_ value of 23.5 μM [28]. A 10-membered ring macrolide, gloeolactone (**55**), was isolated from the cyanobacterium *Gloeotrichia* sp., harvested in Clark Canyon Reservoir (Figure 11). Gloeolactone (**55**) exhibits weak toxicity to brine shrimp. All brine shrimps are dead when the concentration of gloeolactone (**55**) is 125 μg/mL [48]. Phormidolide (**56**) was isolated from the marine cyanobacterium *Phormidium* sp. cultured in Indonesia and was found to exhibit very high toxicity (LC_50_ value of 1.5 μM) in the brine shrimp test [49].

A symmetrical macrolide dimer, cyanolide A (**57**), was obtained from the marine cyanobacterium *Lyngbya bouillonii* collected from Papua New Guinea. Cyanolide A (**57**) displays potent molluscicidal activity against the snail vector *Biomphalaria glabrata* with an LC_50_ value of 1.2 µM. Cyanolide A (**57**) can be used as a new, potent molluscicidal agent to effectively control the spread of schistosomiasis [50]. Its total synthesis has been accomplished [51].

## 5. Other Bioactivity

Cyanobacterium-derived macrolides with rich chemical diversity show various important bioactivities (Table 4). The macrolide biselyngbyaside (**17**), isolated from the marine cyanobacterium *Lyngbya* sp., has been investigated for its effects on osteoclast differentiation and function. Biselyngbyaside (**17**) inhibits RANKL-induced osteoclastogenesis by inhibiting the expression of c-Fos and NFATc1 in mouse monocytic RAW264 cells. Therefore, biselyngbyaside (**17**) is a potentially promising compound with therapeutic and preventive activities against bone-lytic diseases [52]. A toxic cyanobacterial macrolide, debromoaplysiatoxin (**39**), has been found to cause severe cutaneous inflammation in humans and other animals after topical application [25].

A rare 40-membered polyhydroxy macrolide, bastimolide A (**50**), exhibits high selectivity and antimalarial activity against four drug-resistant malaria parasite strains, including TM90-C2A, TM90-C2B, W2 and TM91-C235, with IC_50_ values of 80, 90, 140 and 270 nM, respectively. It has been proven that bastimolide A (**50**) is a potentially promising antimalarial lead compound with high selectivity and antimalarial activity against drug-resistant strains [31]. Malyngolide dimer (**58**) was isolated from the marine cyanobacterium *Lyngbya majuscula* collected from Panama and was shown to exhibit moderate antimalarial activity against chloroquine-resistant *Plasmodium falciparum* (W2) with an IC_50_ value of 19 µM [53].

**Figure 12:**
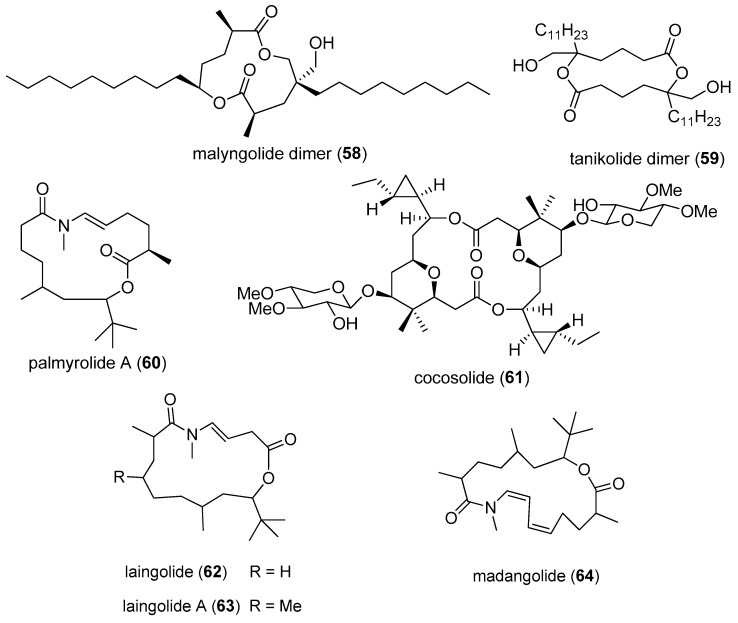
Chemical structures of Compounds **58**–**64**.

A novel SIRT2-selective inhibitor, tanikolide dimer (**59**), was isolated from marine cyanobacterium *Lyngbya majuscula* collected from Madagascar, and it possesses a symmetrical dimer, which has been elucidated by comparison of the natural and synthetic stereoisomers using chiral GC-MS (Figure 12). The tanikolide dimer (**59**) is a potent and selective SIRT2 inhibitor with an IC_50_ value of 176 nM [54].

An unusually stabilized neuroactive macrolide, palmyrolide A(**60**), was isolated, via an assay-based screening program for new neuroactive compounds from cyanobacteria *Leptolyngbya cf.* and *Oscillatoria* spp. harvested in Palmyra Atoll. Palmyrolide A (**60**) contains a rare *N*-methyl enamide and an intriguing *tert*-butyl group, and it can potently inhibit Ca^2+^ oscillations in murine cerebrocortical neuronal cells with an IC_50_ value of 3.70 µM. Moreover, palmyrolide A (**60**) can significantly block the sodium channel activity of neuro-2a cells (IC_50_ value of 5.2 µM) without appreciable cytotoxicity. The above intriguing experimental results suggest that palmyrolide A (**60**) could be a promising drug candidate for further pharmacological exploration [55], and its total synthesis has been completed [56].

A dimeric macrolide, cocosolide (**61**), was isolated from the marine cyanobacterium *Symploca* sp. from Guam, and it strongly inhibits IL-2 production in both T-cell receptor-dependent and independent manners. Both the presence of the sugar moiety and the integrity of the dimeric structure ensure the functionality of cocosolide (**61**). In addition, the total synthesis of cocosolide (**61**) has been accomplished [7].

Three novel nitrogen-containing macrolides, laingolide (**62**) [57], laingolide A (**63**) and madangolide (**64**) [58], have been identified from the marine cyanobacterium *Lyngbya bouillonii* harvested in Laing Island, Papua-New Guinea (Figure 12). The structures of these macrolides (**62–64**) contain a lactone ring of 15, 15 and 17 members, respectively [58].

## 6. Conclusions

Cyanobacteria are rich sources of various natural products with unprecedented pharmacological and biological activities. Up to the end of 2016, a total of 64 macrolide compounds have been isolated from cyanobacteria, including 49 macrolides from marine cyanobacteria and 15 macrolides from terrestrial cyanobacteria. More than half of the cyanobacterium-derived macrolides, a total of 36 compounds, were isolated from the cyanobacterial genus *Lyngbya* species, particularly from *Lyngbya majuscula*. Most of these cyanobacterium-derived macrolides possess several noticeable bioactivities, including antitumor, antibacterial and antimalarial. The overwhelming majority of cyanobacteria derived macrolides (**1**–**51**) display in vitro antitumor activity. Secondary metabolites of cyanobacteria are widely evaluated for their antitumor effects since many metabolites of cyanobacteria have exhibited potent antitumor activities. Some of these macrolides, including tolytoxin (**38**), bastimolide A (**50**) and tanikolide dimer (**59**), exhibited surprisingly strong bioactivity, thus representing potential new drug lead compounds, which are worthy of further research on synthesis and pharmacological activity. The total synthesis of 10 bioactive macrolides, such as cocosolide, has been achieved with a great deal of efforts. The research on the total synthesis of macrolides will promote pharmacologic research and create new opportunities to undertake research in drug discovery, medicine design and large-scale manufacturing. At present, three scholars, including Luesch, Moore and Gerwick, have greatly contributed to the discovery of new macrolides from cyanobacteria. Cyanobacteria have great potentials as sustainable sources for the production of bioactive metabolites because of their rapid growth, genetic tractability and cultivable property. Although cyanobacteria possess the cultivable properties similar to those of microorganisms, cyanobacteria have attracted far less attention than microorganisms. More efforts should be devoted to improving the production of bioactive metabolites in cyanobacteria via cultivation design, metabolic engineering together with efficient isolation. In addition, the programs for drug discovery from cyanobacteria, including the Panama International Cooperative Biodiversity Group (ICGB) program, might facilitate and enhance drug discovery from cyanobacteria. A systematic review on macrolides from cyanobacteria would help establish an effective support system for the discovery and development of cyanobacterium-derived macrolides, and such a support system could also facilitate collection, purification and identification of bioactive macrolides, leading to improve bioactivity assay, synthesis, data analysis and information technology.

## Figures and Tables

**Table 1:** Anti-neoplastic property of cyanobacterium-derived macrolides on different cell lines.

Metabolite	Source	Location	Target Cell Lines	Concentration/Effect	Reference
lyngbyabellin A (**1**)	*Lyngbya majuscula*	Guam	KB cells and LoVo cells	IC_50_ value of 0.03 and 0.50 µg/mL respectively	[6]
lyngbyabellin B (**2**)	*Lyngbya majuscula*	Guam	KB cells and LoVo cells	IC_50_ value of 0.10 and 0.83 µg/mL respectively	[10]
lyngbyabellin E (**3**)	*Lyngbya majuscula*	Papua New Guinea	NCI-H460 human lung tumor and neuro-2a mouse neuroblastoma cells	LC_50_ value of 0.4 and 1.2 µM respectively	[11]
lyngbyabellin F (**4**)	*Lyngbya majuscula*	Papua New Guinea	NCI-H460 human lung tumor and neuro-2a mouse neuroblastoma cells	LC_50_ value of 1.0 and 1.8 µM respectively	[11]
lyngbyabellin G (**5**)	*Lyngbya majuscula*	Papua New Guinea	NCI-H460 human lung tumor and neuro-2a mouse neuroblastoma cells	LC_50_ value of 2.2 and 4.8 µM respectively	[11]
lyngbyabellin H (**6**)	*Lyngbya majuscula*	Papua New Guinea	NCI-H460 human lung tumor and neuro-2a mouse neuroblastoma cells	LC_50_ value of 0.2 and 1.4 µM respectively	[11]
lyngbyabellin I (**7**)	*Lyngbya majuscula*	Papua New Guinea	NCI-H460 human lung tumor and neuro-2a mouse neuroblastoma cells	LC_50_ value of 1.0 and 0.7 µM respectively	[11]
lyngbyabellin N (**10**)	*Moorea bouillonii*	Palmyra Atoll, USA	H-460 human lung carcinoma and HCT116 colon cancer cell lines	IC_50_ value of 0.0048–1.8 µM and 15 µM respectively	[12]
lyngbyaloside B (**13**)	*Lyngbya* sp.	Palau	KB cells and LoVo cells	IC_50_ value of 4.3 and 15 µM respectively	[13]
2-*epi*-lyngbyalosid (**14**)	*Lyngbya bouillonii*	Apra Harbor, Guam	HT29 colorectal adenocarcinoma and HeLa cells	IC_50_ value of 38 and 33 µM respectively	[14]
18*E*-lyngbyaloside C (**15**)	*Lyngbya bouillonii*	Apra Harbor, Guam	HT29 colorectal adenocarcinoma and HeLa cells	IC_50_ value of 13 and 9.3 µM respectively	[14]
18*Z*-lyngbyaloside C (**16**)	*Lyngbya bouillonii*	Apra Harbor, Guam	HT29 colorectal adenocarcinoma and HeLa cells	IC_50_ value of >100 µM and 53 µM respectively	[14]
biselyngbyaside (**17**)	*Lyngbya* sp.	Tokunoshima Island, Japan	HeLa S_3_ cells	IC_50_ value of 0.1 μg/mL	[15]
biselyngbyaside B (**18**)	*Lyngbya* sp.	Tokunoshima Island, Japan	HeLa S_3_ cells and HL60 cells	IC_50_ value of 3.5 and 0.82 µM respectively	[16]
biselyngbyaside E (**21**)	*Lyngbya* sp.	Ishigaki Island, Japan	HeLa and HL60 cells	IC_50_ value of 0.19 and 0.071 µM respectively	[17]
biselyngbyaside F (**22**)	*Lyngbya* sp.	Ishigaki Island, Japan	HeLa and HL60 cells	IC_50_ value of 3.1 and 0.66 µM respectively	[17]
biselyngbyolide A (**23**)	*Lyngbya* sp.	Tokunoshima Island, Japan	HeLa S_3_ cells and HL60 cells	IC_50_ value of 0.22 and 0.027 µM respectively	[18]
biselyngbyolide B (**24**)	*Lyngbya* sp.	Ishigaki Island, Japan	HeLa S_3_ cells and HL60 cells	IC_50_ value of 0.028 and 0.0027 µM respectively	[19]
caylobolide A (**25**)	*Lyngbya majuscula*	Bahamian	human colon tumor cells HCT 116	IC_50_ values of 9.9 µM	[20]
caylobolide B (**26**)	*Phormidium* spp.	Florida USA	HT29 colorectal adenocarcinoma and HeLa cervical carcinoma cells	IC_50_ value of 4.5 and 12.2 µM respectively	[21]
swinholide A (**27**)	*Symploca cf.* sp.	Fiji	several cancer cell lines	IC_50_ values of 0.37 nM–1.0 µM	[22]
ankaraholide A (**28**)	*Geitlerinema* sp.	Madagascar	NCI-H460, Neuro-2a cells and MDA-MB-435 cells	IC_50_ value of 119, 262 and 8.9 nM respectively	[22]
scytophycin A (**30**)	*Scytonema pseudohofmanni*	Oahu, Hawaii	human carcinoma of nasopharynx Cell (KB cells)	IC_50_ value of 1 ng/mL	[23]
scytophycin B (**31**)	*Scytonema pseudohofmanni*	Oahu, Hawaii	KB cells	IC_50_ value of 1 ng/mL	[23]
scytophycins C-E (**32**–**34**)	*Scytonema pseudohofmanni*	Oahu, Hawaii	KB cells	IC_50_ value of 10–100 ng/mL	[23]
6-hydroxyscytophycin B (**35**)	*Scytonema mirabile*	cultured	KB cells and LoVo cells	MICs of 1–5 and 10–50 ng/mL respectively	[23]
19-*O*-demethylscytophycin C (**36**)	*Scytonema burmanicurn*	cultured	KB cells and LoVo cells	MICs of 1–5 and 10–50 ng/mL respectively	[23]
6-hydroxy-7-*O*-methylscytophycin E (**37**)	*Scytonema ocellatum*	cultured	KB cells and LoVo cells	MICs of 1–5 and 10–50 ng/mL respectively	[23]
tolytoxin (**38**)	*Tolypothrix conglutinata* var. *colorata*	Fanning Island	KB cells and LoVo cells	IC_50_ value of 8.4 and 5.3 nM respectively	[24]
debromoaplysiatoxin (**39**)	*Lyngbya majuscula*	Marshall Islands	P-388 lymphocytic mouse leukemia	weak	[25]
lyngbouilloside (**44**)	*Lyngbya bouillonii*	Papua New Guinea	neuroblastoma cells	IC_50_ value of 17 µM	[26]
koshikalide (**45**)	*Lyngbya* sp.	Mie Prefecture	HeLa S_3_ cells	IC_50_ value of 42 µg/mL	[27]
sanctolide A (**46**)	*Oscillatoria sancta*	cultured	HT-29 and MDA-MB-435 cell lines	nd ^a^	[28]
acutiphycin (**47**)	*Oscillatoria acutissima*	Manoa Valley Oahu, Hawaii	KB cells and NIH/3T3 cells	ED_50_ < 1 µg/mL	[29]
20,21-didehydroacutiphycin (**48**)	*Oscillatoria acutissima*	Manoa Valley Oahu, Hawaii	KB cells and NIH/3T3 cells	ED_50_ < 1 µg/mL	[29]
polycavernoside D (**49**)	*Okeania* sp.	Puerto Rican	H-460 human lung cancer cell lines	EC_50_ value of 2.5 µM	[30]
bastimolide A (**50**)	*Okeania hirsuta*	Panama	Vero cells	IC_50_ value of 2.1 µM	[31]
nuiapolide (**51**)	colonial cyanobacterium (071905-NII-01)	Hawaii	Jurkat cells and cancerous T lymphocytes	anti-chemotactic activity	[32]

**^a^** Not determined.

**Table 2:** Antibacterial and antifungal macrolides.

Metabolite	Source	Location	Target	Concentration/Effect	Reference
6-hydroxyscytophycin B (**35**)	*Scytonema mirabile*	cultured	Fungus (1) *Aspergillus oryzae* (2) *Candida albicans* (3) *Penicillium notatum* (4) *Saccharomyces cerevisiae*	nd ^a^	[23]
19-*O*-demethylscytophycin C (**36**)	*Scytonema burmanicurn*	cultured	Fungus (1) *Aspergillus oryzae* (2) *Candida albicans* (3) *Penicillium notatum* (4) *Saccharomyces cerevisiae*	nd ^a^	[23]
6-hydroxy-7-*O*-methylscytophycin E (**37**)	*Scytonema ocellatum*	cultured	Fungus (1) *Aspergillus oryzae* (2) *Candida albicans* (3) *Penicillium notatum* (4) *Saccharomyces cerevisiae*	nd ^a^	[23]
tolytoxin (**38**)	*Tolypothrix conglutinata* var. *colorata*	Fanning Island	Fungi *Penicillium notatum* and *Rhizoctonia solani* 1165	MIC value of 0.25 nM respectively	[24]
7-OMe-scytophycin B (**52**)	*Anabaena* sp.	cultured	Fungus *Candida albicans* HAMBI 484 and *Candida guilliermondii* HAMBI 257	MIC values of 0.40 and 0.80 mM respectively; IC_50_ value of 0.19 and 0.23 µM respectively	[45]
amantelide A (**53**)	Oscillatoriales	Guam	Fungi *Lindra thalassiae* and *Fusarium* sp.	totally inhibited of 62.5 μg/mL	[46]
amantelide B (**54**)	Oscillatoriales	Guam	Fungus *Dendryphiella salina*	totally inhibited of 6.25 μg/mL	[46]

**^a^** Not determined.

**Table 3:** Effects of cyanobacterium-derived macrolides on animals.

Metabolite	Source	Location	Target Fauna	Impacts	Reference
lyngbyabellin A (**1**)	*Lyngbya majuscula*	Guam	mice	LD_50_ value of 1.2–1.5 mg/kg	[6]
tolytoxin (**38**)	*Scytonema pseudohofmanni*	cultured	mice	LD_50_ value of 1.5 mg/kg	[24]
sanctolide A (**48**)	*Oscillatoria sancta*	cultured	brine shrimp	LD_50_ value of 23.5 μM	[28]
gloeolactone (**55**)	*Gloeotrichia* sp.	Clark Canyon Reservoir	brine shrimp	100% killed at 125 µg/mL	[48]
phormidolide (**56**)	*Phormidium* sp.	Sulawesi, Indonesia	brine shrimp	LD_50_ value of 1.5 μM	[49]
cyanolide A (**57**)	*Lyngbya bouillonii*	Papua New Guinea	snail vector *Biomphalaria glabrata*	LD_50_ value of 1.2 μM	[50]

**Table 4:** Other bioactivity of cyanobacterium-derived macrolides.

Metabolite	Source	Location	Biological Activity	Reference
biselyngbyaside (**17**)	*Lyngbya* sp.	Okinawa Prefecture Japan	osteoclast differentiation and function	[52]
debromoaplysiatoxin (**39**)	*Lyngbya majuscula*	Enewetak Atoll, Marshall Islands	produce an irritant pustular folliculitis in humans and cause a severe cutaneous inflammatory reaction in the rabbit and in hairless mice	[25]
bastimolide A (**50**)	*Okeania hirsuta*	Caribbean coast of Panama	*Plasmodium falciparum* TM90-C2A, TM90-C2B, W2, TM91-C235 (IC_50_ values of 80, 90, 140 and 270 nM respectively)	[31]
malyngolide dimer (**58**)	*Lyngbya majuscula*	Panama	*Plasmodium falciparum* (IC_50_ values of 19 µM)	[53]
tanikolide dimer (**59**)	*Lyngbya majuscula*	Madagascar	SIRT2 (IC_50_ = 176 nM to 2.4 µM)	[54]
palmyrolide A (**60**)	*Leptolyngbya cf. Oscillatoria* sp.	Palmyra Atoll	suppression of calcium influx in cerebrocortical neurons (IC_50_ value of 3.70 µM) sodium channel blocking activity in neuro-2a cells (IC_50_ value of 5.2 µM)	[55]
cocosolide (**61**)	*Symploca* sp.	Guam	inhibition of IL-2 production and T-cell proliferation	[7]

## References

[1] Capper A., Erickson A.A., Ritson-Williams R., Becerro M.A., Arthur K.A., Paul V.J. (2016). Palatability and chemical defences of benthic cyanobacteria to a suite of herbivores. J. Exp. Mar. Biol. Ecol..

[2] Tan L.T. (2007). Bioactive natural products from marine cyanobacteria for drug discovery. Phytochemistry.

[3] Napolitano J.G., Daranas A.H., Norte M., Fernández J.J. (2009). Marine macrolides, a promising source of antitumor compounds. Anti-Cancer Agent Med. Chem..

[4] Kollár P., Rajchard J., Balounová Z., Pazourek J. (2014). Marine natural products: bryostatins in preclinical and clinical studies. Pharm. Biol..

[5] Belakhov V.V., Garabadzhiu A.V. (2015). Polyene macrolide antibiotics: mechanisms of inactivation, ways of stabilization, and methods of disposal of unusable drugs (review). Russ. J. Gen. Chem..

[6] Luesch H., Yoshida W.Y., Moore R.E., Paul V.J., Mooberry S.L. (2000). Isolation, structure determination, and biological activity of lyngbyabellin A from the marine cyanobacterium *Lyngbya majuscula*. J. Nat. Prod..

[7] Gunasekera S.P., Li Y., Ratnayake R., Luo D.M., Lo J., Reibenspies J.H., Xu Z.S., Clare-Salzler M.J., Ye T., Paul V.J., Luesch H. (2016). Discovery, total synthesis and key structural elements for the immunosuppressive activity of cocosolide, a symmetrical glycosylated macrolide dimer from marine cyanobacteria. Chem. Eur. J..

[8] Klein D., Braekman J.C., Daloze D., Hoffmann L., Demoulin V. (1997). Lyngbyaloside, a novel 2,3,4-Tri-*O*-methyl-6-deoxy-r-mannopyranoside macrolide from *Lyngbya bouillonii* (Cyanobacteria). J. Nat. Prod..

[9] DeVita V.T.J., Rosenberg S.A. (2012). Two hundred years of cancer research. N. Engl. J. Med..

[10] Luesch H., Yoshida W.Y., Moore R.E., Paul V.J. (2000). Isolation and structure of the cytotoxin lyngbyabellin B and absolute configuration of lyngbyapeptin A from the marine cyanobacterium *Lyngbya majuscula*. J. Nat. Prod..

[11] Han B.N., McPhail K.L., Gross H., Goeger D.E., Mooberry S.L., Gerwick W.H. (2005). Isolation and structure of five lyngbyabellin derivatives from a Papua New Guinea collection of the marine cyanobacterium *Lyngbya majuscula*. Tetrahedron.

[12] Choi H., Mevers E., Byrum T., Valeriote F.A., Gerwick W.H. (2012). Lyngbyabellins K–N from Two Palmyra Atoll Collections of the marine cyanobacterium *Moorea bouillonii*. Eur. J. Org. Chem..

[13] Luesch H., Yoshida W.Y., Harrigan G.G., Doom J.P., Moore R.E., Paul V.J. (2002). Lyngbyaloside B, a new glycoside macrolide from a palauan marine cyanobacterium, *Lyngbya* sp.. J. Nat. Prod..

[14] Matthew S., Salvador L.A., Schupp P.J., Paul V.J., Luesch H. (2010). Cytotoxic halogenated macrolides and modified peptides from the apratoxin-producing marine cyanobacterium *Lyngbya bouillonii* from Guam. J. Nat. Prod..

[15] Teruya T., Sasaki H., Kitamura K., Nakayama T., Suenaga K. (2009). Biselyngbyaside, a macrolide glycoside from marine cyanobacterium *Lyngbya* sp.. Org. Lett..

[16] Morita M., Ohno O., Teruya T., Yamori T., Inuzuka T., Suenaga K. (2012). Isolation and structures of biselyngbyasides B, C, and D from the marine cyanobacterium *Lyngbya* sp., and the biological activities of biselyngbyasides. Tetrahedron.

[17] Watanabe A., Ohno O., Morita M., Inuzuka T., Suenaga K. (2015). Structures and biological activities of novel biselyngbyaside analogs isolated from the marine cyanobacterium *Lyngbya* sp.. Bull. Chem. Soc. Jpn..

[18] Morita M., Ohno O., Suenaga K. (2012). Biselyngbyolide A, a novel cytotoxic macrolide from the marine cyanobacterium *Lyngbya* sp.. Chem. Lett..

[19] Ohno O., Watanabe A., Morita M., Suenaga K. (2014). Biselyngbyolide B, a novel ER stress-inducer isolated from the marine cyanobacterium *Lyngbya* sp.. Chem. Lett..

[20] MacMillan J.B., Molinski T.F. (2002). Caylobolide A, a unique 36-membered macrolactone from a Bahamian *Lyngbya majuscula*. Org. Lett..

[21] Salvador L.A., Paul V.J., Luesch H. (2010). Caylobolide B, a macrolactone from symplostatin 1-producing marine cyanobacteria *phormidium* spp. from Florida. J. Nat. Prod..

[22] Andrianasolo E.H., Gross H., Goeger D., Musafija-Girt M., McPhail K., Leal R.M., Mooberry S.L., Gerwick W.H. (2005). Isolation of swinholide A and related glycosylated derivatives from two field collections of marine cyanobacteria. Org. Lett..

[23] Carmeli S., Moore R.E., Patterson G.M.L. (1990). Tolytoxin and new scytophycins from three species of *Scytonema*. J. Nat. Prod..

[24] Patterson G.M.L., Carmeli S. (1992). Biological effects of tolytoxin (6-hydroxy-7-*O*-methyl-scytophycin b), a potent bioactive metabolite from cyanobacteria. Arch. Microbiol..

[25] Mynderse J.S., Moore R.E., Kashiwagi M., Norton T.R. (1977). Antileukemia activity in the Osciliatoriaceae: Isolation of debromoaplysiatoxin from *Lyngbya*. Science.

[26] Tan L.T., Márquez B.L., Gerwick W.H. (2002). Lyngbouilloside, a novel glycosidic macrolide from the marine cyanobacterium *Lyngbya bouillonii*. J. Nat. Prod..

[27] Iwasaki A., Teruya T., Suenaga K. (2010). Isolation and structure of koshikalide, a 14-membered macrolide from the marine cyanobacterium *Lyngbya* sp.. Tetrahedron Lett..

[28] Kang H.S., Krunic A., Orjala J. (2012). Sanctolide A, a 14-membered PK-NRP hybrid macrolide from the cultured cyanobacterium *Oscillatoria sancta* (SAG 74.79). Tetrahedron Lett..

[29] Barchi J.J., Moore R.E., Patterson G.M.L. (1984). Acutiphycin and 20,21-didehydroacutiphycin, new antineoplastic agents from the cyanophyte *Oscillatoria acutissima*. J. Am. Chem. Soc..

[30] Navarro G., Cummings S., Lee J., Moss N., Glukhov E., Valeriote F.A., Gerwick L., Gerwick W.H. (2015). Isolation of polycavernoside D from a marine cyanobacterium. Environ. Sci. Technol. Lett..

[31] Shao C.L., Linington R.G., Balunas M.J., Centeno A., Boudreau P., Zhang C., Engene N., Spadafora C., Mutka T.S., Kyle D.E., Gerwick L., Wang C.Y., Gerwick W.H. (2015). Bastimolide A, a potent antimalarial polyhydroxy macrolide from the marine cyanobacterium *Okeania hirsute*. J. Org. Chem..

[32] Mori S., Williams H., Cagle D., Karanovich K.F., Horgen D., Smith R., Watanabe C.M.H. (2015). Macrolactone nuiapolide, isolated from a Hawaiian marine cyanobacterium, exhibits anti-chemotactic activity. Mar. Drugs.

[33] Fuwa H., Yamagata N.Y., Okuaki Y.T., Ogata Y.Y., Saito A., Sasaki M. (2016). total synthesis and complete stereostructure of a marine macrolide glycoside, (-)-lyngbyaloside B. Chem. Eur. J..

[34] Chang C., Stefan E., Taylo R.E. (2015). Total synthesis and structural reassignment of lyngbyaloside C highlighted by intermolecular ketene esterification. Chem. Eur. J..

[35] Chandrasekhar S., Rajesh G., Naresh T. (2013). Enantioselective synthesis of the C5–C23 segment of biselyngbyaside. Tetrahedron Lett..

[36] Yadav J.S., Swapnil N., Venkatesh M., Prasad A.R. (2014). Studies directed toward the synthesis of caylobolide A: convergent synthesis of C21–C40 subunit. Tetrahedron Lett..

[37] Ishibashi M., Moore R.E., Patterson G.M.L. (1986). Scytophycins, cytotoxic and antimycotic agents from the cyanophyte *Scytonema pseudohofmanni*. J. Org. Chem..

[38] Cui J., Watanabe T., Shibasaki M. (2016). Catalytic asymmetric synthesis of key intermediate for scytophycin C. Tetrahedron Lett..

[39] Moore R.E., Scheuer P.J. (1981). Constituents of blue-green algae. Marine Natural Products.

[40] Solomon A.E., Stoughton R.B. (1978). Dermatitis from purified sea algae toxin (debromoaplysiatoxin). Arch. Dermatol..

[41] Mynderse J.S., Moore R.E. (1978). Toxins from Blue-Green algae: Structures ofoscillatoxin A and three related bromine-containing toxins. J. Org. Chem..

[42] Kunifuda K., Iwasaki A., Nagamoto M., Suenaga K. (2016). Total synthesis and absolute configuration of koshikalide. Tetrahedron Lett..

[43] Yadav J.S., Suresh B., Srihari P. (2015). Stereoselective total synthesis of the marine macrolide sanctolide A. Eur. J. Org. Chem..

[44] Frayman K., Robinson P. (2013). Macrolide therapy in cystic fibrosis: New developments in clinical use. Clin. Investig..

[45] Shishido T.K., Humisto A., Jokela J., Liu L.W., Wahlsten M., Tamrakar A., Fewer D.P., Permi P., Andreote A.P.D., Fiore M.F., Sivonen K. (2015). Antifungal compounds from cyanobacteria. Mar. Drugs.

[46] Salvador-Reyes L.A., Sneed J., Paul V.J., Luesch H. (2015). Amantelides A and B, polyhydroxylated macrolides with differential broad-spectrum cytotoxicity from a guamanian marine cyanobacterium. J. Nat. Prod..

[47] Wood R. (2016). Acute animal and human poisonings from cyanotoxin exposure—A review of the literature. Environ. Int..

[48] Stierle D.B., Stierle A.A., Bugni T., Loewen G. (1998). Gloeolactone, a new epoxy lactone from a blue-green alga. J. Nat. Prod..

[49] Williamson R.T., Boulanger A., Vulpanovici A., Roberts M.A., Gerwick W.H. (2002). Structure and absolute stereochemistry of phormidolide, a new toxic metabolite from the marine cyanobacterium *phormidium* sp.. J. Org. Chem..

[50] Pereira A.R., McCue C.F., Gerwick W.H. (2010). Cyanolide A, a glycosidic macrolide with potent molluscicidal activity from the Papua New Guinea cyanobacterium *Lyngbya bouillonii*. J. Nat. Prod..

[51] Bates R.W., Lek T.G. (2014). A synthesis of cyanolide A by intramolecular oxa-michael addition. Synthesis.

[52] Yonezawa T., Mase N., Sasaki H., Teruya T., Hasegawa S., Cha B.Y., Yagasaki K., Suenaga K., Nagai K., Woo J.T. (2012). Biselyngbyaside, isolated from marine cyanobacteria, inhibits osteoclastogenesis and induces apoptosis in mature osteoclasts. J. Cell. Biochem..

[53] Gutiérrez M., Tidgewell K., Capson T.L., Engene N., Almanza A., Schemies J., Jung M., Gerwick W.H. (2010). Malyngolide Dimer, a bioactive symmetric cyclodepside from the panamanian marine cyanobacterium *Lyngbya majuscula*. J. Nat. Prod..

[54] Gutierrez M., Andrianasolo E.H., Shin W.K., Goeger D.E., Yokochi A., Schemies J., Jung M., France D., Cornell-Kennon S., Lee E. (2009). Structural and synthetic investigations of tanikolide dimer, a SIRT2 selective inhibitor, and tanikolideseco-acid from the Madagascar marine cyanobacterium *Lyngbya majuscula*. J. Org. Chem..

[55] Pereira A.R., Cao Z.Y., Engene N., Soria-Mercado I.E., Murray T.F., Gerwick W.H. (2010). Palmyrolide A, an Unusually Stabilized Neuroactive Macrolide from Palmyra Atoll Cyanobacteria. Org. Lett..

[56] Sudhakar G., Reddy K.J., Nanubolu J.B. (2010). Total synthesis of palmyrolide A and its 5,7-*epi* isomers. Org. Lett..

[57] Klein D., Braekman J.C., Daloze D. (1996). Laingolide, a novel 15-membered macrolide from *Lyngbya bouillonii* (cyanophyceae). Tetrahedron Lett..

[58] Klein D., Braekman J.C., Daloze D., Hoffmann L., Castillo G., Demoulin V. (1999). Madangolide and laingolide A, two novel macrolides from *Lyngbya bouillonii* (Cyanobacteria). J. Nat. Prod..

